# Association of circulating tumor DNA from the cerebrospinal fluid with high‐risk CNS involvement in patients with diffuse large B‐cell lymphoma

**DOI:** 10.1002/ctm2.236

**Published:** 2021-01-15

**Authors:** Xiaoxiao Wang, Yan Gao, Changguo Shan, Mingyao Lai, Haixia He, Bing Bai, Liqin Ping, Qixiang Rong, Ruyu Ai, Lei Wen, Zhaoming Zhou, Ruoying Yu, Qiuxiang Ou, Xue Wu, Xiaoxia Wang, Yang W. Shao, Linbo Cai, Huiqiang Huang

**Affiliations:** ^1^ State Key Laboratory of Oncology in South China Collaborative Innovation Center for Cancer Medicine Guangzhou China; ^2^ Department of Medical Oncology Sun Yat‐sen University Cancer Center Guangzhou China; ^3^ Department of Medical Oncology Guangdong Sanjiu Brain Hospital Guangzhou China; ^4^ Translational Medicine Research Institute Geneseeq Technology Inc. Toronto Ontario Canada; ^5^ Nanjing Geneseeq Technology Inc. Nanjing China; ^6^ School of Public Health Nanjing Medical University Nanjing China

Dear Editor,

Central nervous system (CNS) involvement in diffuse large B‐cell lymphoma (DLBCL) patients correlates with dismal outcomes, and the detection sensitivity of conventional diagnosis of lymphoma is restricted.^1^, ^2^, ^3^, ^4^ Circulating tumor DNA from cerebrospinal fluid (CSF‐ctDNA) has played an important part in the application of liquid biopsy for patients with CNS cancers.^5^ In this study, we provided new insights into feasibility of CSF‐derived biomarkers for CNS relapse diagnosis in DLBCL patients. In clinical setting, the diagnosis of CNS involvement is based on several clinical risk factors including individual international prognostic index (IPI), number of extranodal involvement (testicular/adrenal/kidney), and serum lactate dehydrogenase (LDH).^6^ CNS‐IPI, which is a six‐risk‐factor model developed by a German group (five IPI factors with kidney/adrenal involvement) for CNS diagnosis, has been validated and proved to be useful in clinical settings.^7^ Other reported biological risk factors for CNS involvement included *MYC* gene rearrangements or *MYC (MYC proto‐oncogene)* and *BCL2 (B‐cell lymphoma 2)* dual translocations.^8^, ^9^

To assess the correlation between CSF‐ctDNA and CNS involvement in DLBCL, targeted mutational profiling was performed on CSF‐ and plasma‐derived ctDNA together with matched systemic tumor tissues in 67 DLBCL patients clinically diagnosed as high risk for CNS involvement (Figure S1, Table S1). Genomic landscape of this DLBCL cohort in systemic tumor tissue is shown in Figure 1A. Considering both single nucleotide variant (SNV) and copy number variant (CNV), commonly mutated genes cohort included *Pim‐1 proto‐oncogene* (*PIM1*, 37.3%), *lysine methyltransferase 2D* (*KMT2D*,33.3%), *BCL2* (27.5%), *myeloid differentiation primary response 88* (*MYD88*, 27.5%), *B‐cell translocation gene 2* (*BTG2*, 23.5%), and *tumor protein p53* (*TP53*, 23.5%). Majority of altered genes were involved in four important pathways including epigenetic regulation (*KMT2D*, *CREB‐binding protein/CREBBP*), BCR (*breakpoint cluster region gene*) and NF‐kB (*Nuclear factor‐kappa B)* signaling pathway (*Bruton tyrosine kinase*/*BTK*, *MYD88*), apoptosis/cell cycle (*BTG2*, *ETS variant transcription factor 6/ETV6)*, and immunity (*dual specificity phosphatase 2/DUSP2, CD58*,). SNV was the most frequently observed type of alteration, whereas CNV had the tendency to be enriched in patients with lower CNS‐IPI including *BCL2* and *MALT1 (mucosa‐associated lymphoid tissue lymphoma translocation protein 1)* amplification. Interestingly, cell‐free DNA (cfDNA) concentration in CSF‐in DLBCL patients showed a trend of increase from low to high level CNS‐IPI. By dividing patients into 0‐3 and 4‐6 CNS‐IPI, we observed a significant elevation in CSF‐cfDNA concentration, suggesting CSF‐cfDNA might be an indication of CNS involvement (Figure 1B). Plasma‐cfDNA was relatively low in patients with level 0‐1 CNS‐IPI compared to level 2‐6 CNS‐IPI. However, no significantly difference was found between two subgroups (Figure 1C).

**FIGURE 1:**
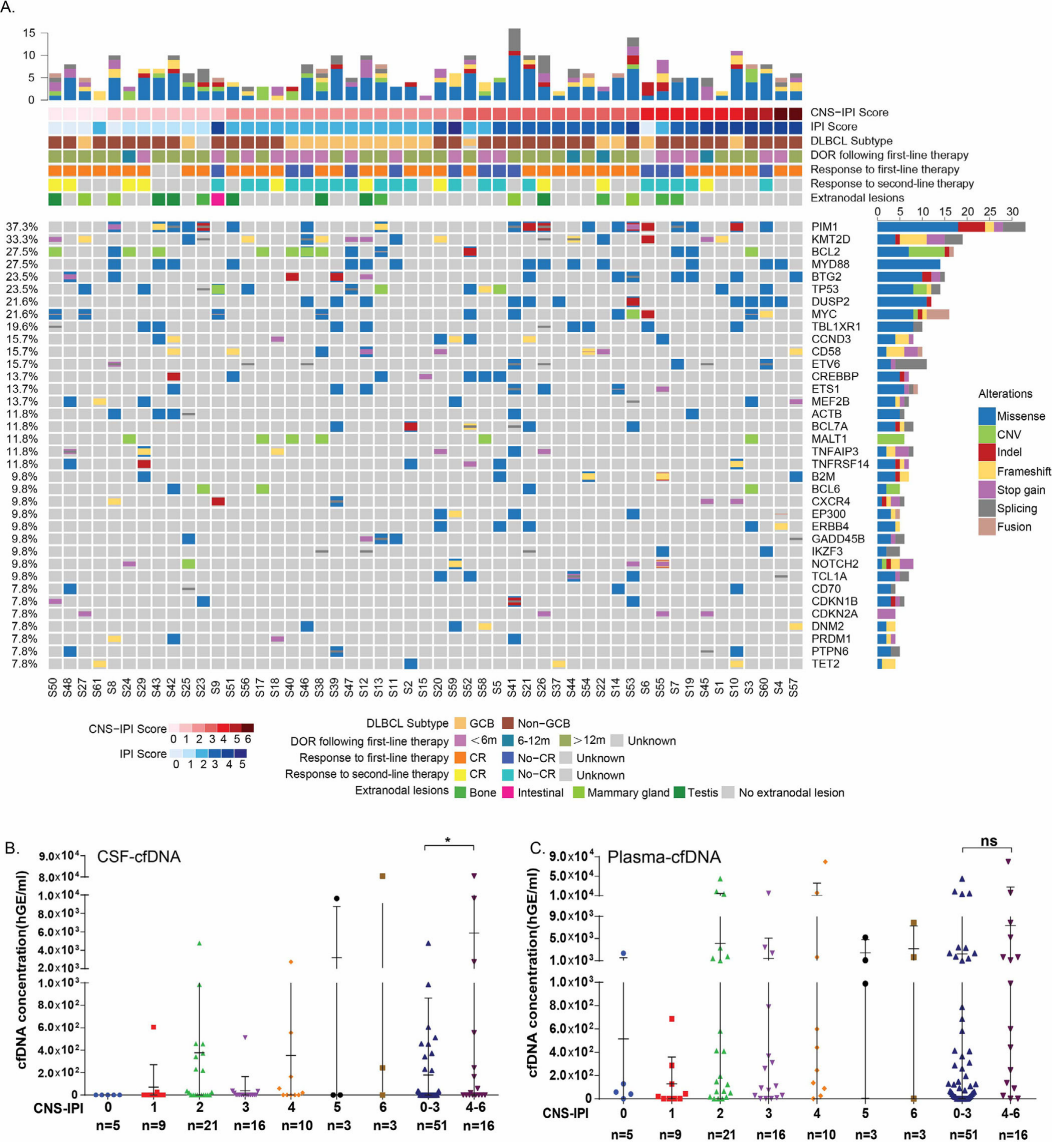
**Increased CSF‐cfDNA concentration correlated with high CNS‐IPI**. (A) Genomic landscape of patients with DLBCL in systemic tumor tissue. Clinical information was indicated by bars on top. Each column represented one patient. (B) Distribution of plasma‐cfDNA and CSF‐cfDNA concentration in DLBCL patients with 0‐6 CNS‐IPI. *P < .05. ns: not significant

In the 20 CSF‐cfDNA‐positive patients, a comparison of gene alterations (GAs) in matched systemic tumor tissue, CSF, and plasma was performed (Figure 2A). Despite the mean allele frequency of GAs was significantly higher in tumor tissue, there was still unique GAs identified in CSF and plasma that could be the potential feature for high‐risk CNS (Figure 2B).The total numbers of identified GAs were 224 GAs in tumor tissue, 134GAs in CSF, and 153GAs in plasma, respectively. Eighty‐six shared GAs (consistency with tissue: 38.39%) were found between tumor tissue and CSF, whereas 95 (consistency with tissue: 42.41%) were found between tumor tissue and plasma (Figure 2C). Interestingly, there were 48 GAs that exclusively found in CSF compared to GAs in tissue, including 24 GAs only in CSF and 24 GAs shared by plasma.

**FIGURE 2:**
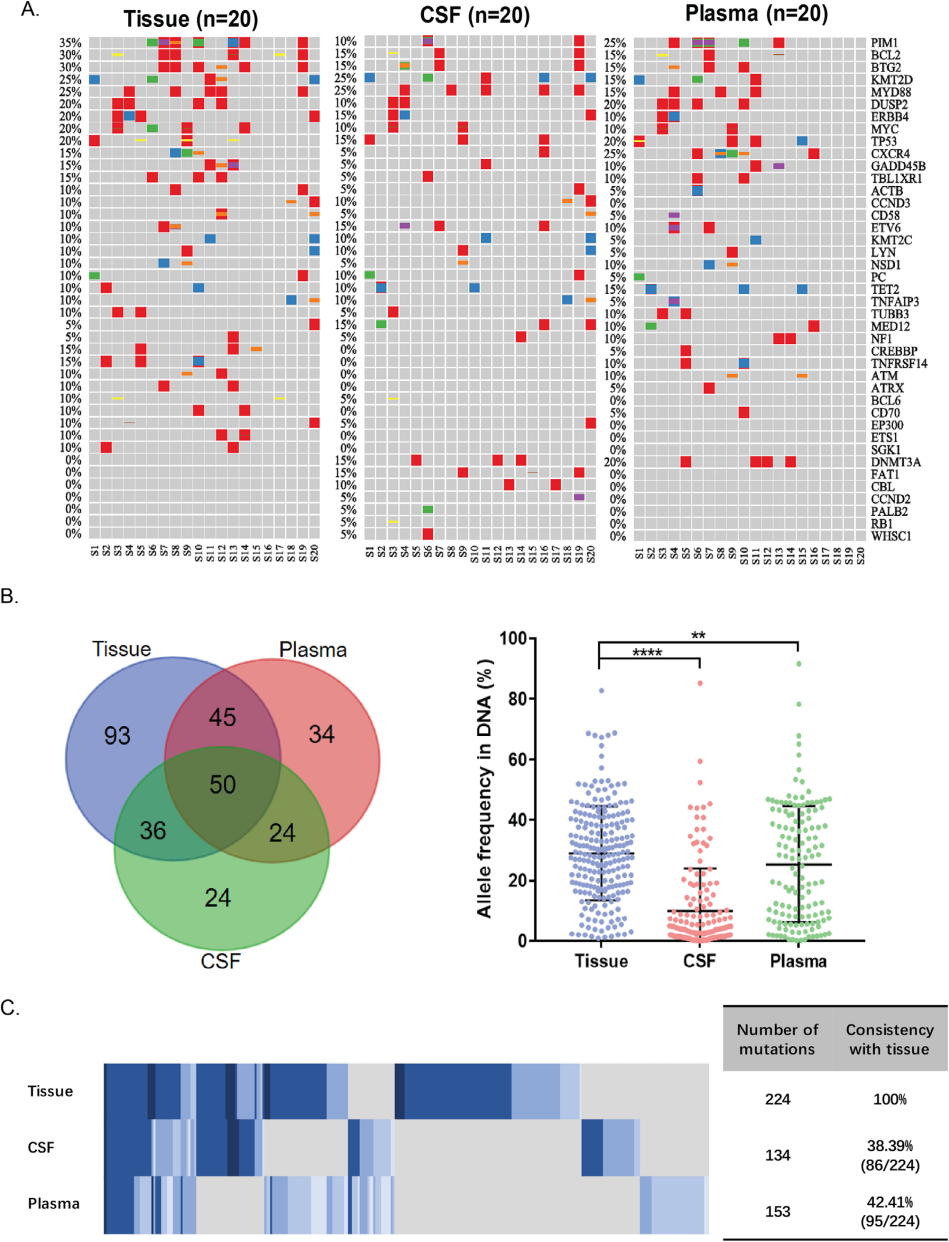
**CSF‐unique and non‐CSF‐unique gene alterations in patients with DLBCL**. (A) Distribution of gene alterations identified in DLBCL patients with matched systemic tumor tissue, CSF, and plasma. Each column represented one patient. (B) Numbers of shared mutations and unique mutations identified in matched systemic tumor tissue, CSF, and plasma. The distribution and mean allele frequency in DNA were shown. (C) Consistency of mutations identified between different sample type

Compared to GAs only found in tumor tissue (93 genes, Figure 2B), 48 CSF‐specific GAs of DLBCL were enriched in apoptosis/cell cycle related pathway and immunity‐related pathway (Figure 3A). To find out whether 48 GAs were indeed CNS related, we employed a cohort of 10 DLBCL patients with primary CNS tumors (PCNSL). Gene alterations from both brain tumor tissue and CSF of PCNSL patients were screened. However, only one GA from the 48 GAs, *BTG2* S31N, was found in the PCNSL cohort.

**FIGURE 3:**
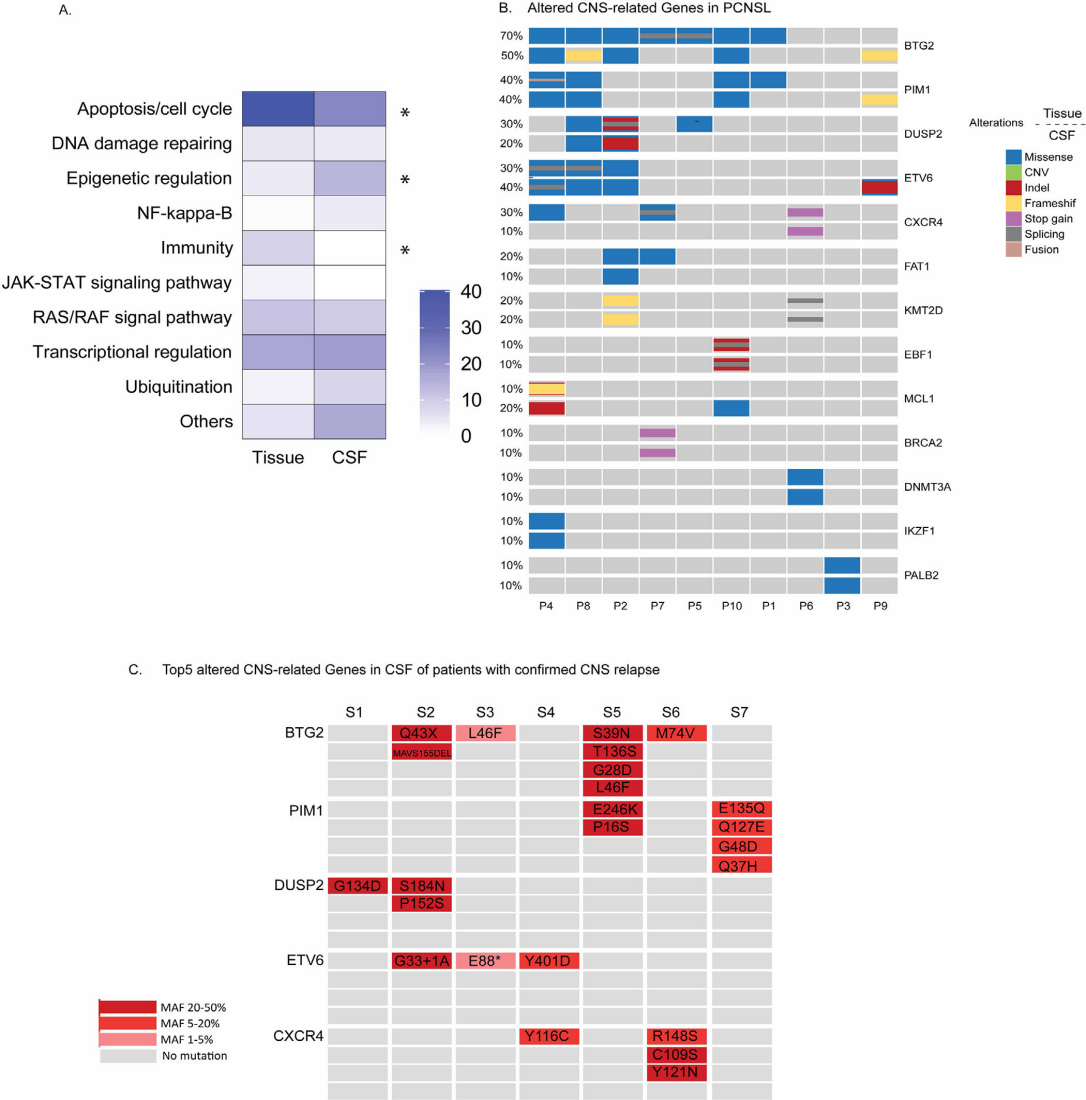
**Altered CNS‐related genes in CSF**. (A) Pathway analysis of genes identified only in CSF and compared to genes identified only in tumor tissue. *P < 0.05. (B) Top altered CNS‐related genes in patient with PCNSL. Each column represented one patient. Frequency of gene alteration in tumor tissue (upper) was compared with frequency of gene alteration in CSF (lower). (C) Alteration in top five CNS‐related genes in MRI/FCM confirmed cases. Mean allele frequency of each mutation was indicated by color

Then we look into the CSF signature of high‐risk DLBCL in the gene level rather than mutation level. Here, we referred the genes with alterations found exclusively in CSF of high‐risk DLBCL as CSF‐CNS genes. As shown in Figure 3B, 13 altered CSF‐CNS genes were identified in both brain tumor tissue and CSF of PCNSL patients. The incident of *BTG2* alterations displayed highest frequency compared to other genes, which accounted for 70% of brain tumor tissue and 50% of CSF. Among 13 altered CSF‐CNS genes shared by PCNSL cohort, five of them (*BTG2*, *PIM1*, *DUSP2*, *ETV6*, *C‐X‐C motif chemokine receptor 4/CXCR4*) were identified in more than 20% of total PCNSL cases. Meanwhile, 31 DLBCL patients with high risk for CNS had at least one alteration in the *BTG2*, *PIM1*, *DUSP2*, *ETV6*, and *CXCR4* genes from CSF and/or plasma, including 14 patients with level4‐6 CNS‐IPI. Ten high‐risk DLBCL patients (nine were CSF‐cfDNA‐positive) were later confirmed CNS involvement by magnetic resonance imaging (MRI) or flow cytometry (FCM). Among them, seven patients were identified with multiple alterations of the five CSF‐CNS genes in CSF (Figure 3C). Moreover, we compared alterations in five CSF‐CNS genes from systemic tumor tissue of DLBCL patients in high‐risk (HR) group with an additional dataset of 40 patients clinically diagnosed as low risk (LR) for CNS relapse. The mutation status of five CSF‐CNS genes was comparable between HR and LR group in tumor tissue. This partially explained that the mutational difference in the five CSF‐CNS genes between HR and LR might be restricted to brain tumor and CSF‐specific (Figure S2A and B). The molecular landscapes in tumor tissue of both DLBCL cohorts with different CNS risk were also similar except MCL1 and CD70 genes (Figure S2C).

Limitations of this study included the lack of CSF and plasma samples from low‐risk group to assess the status of CSF‐CNS genes. Close monitoring of CNS relapse in the high‐risk cohort will be also of great help to further validate our observation. Potential blood contamination or normal cfDNA in the CSF sample might also have some impact on the result interpretation.

In summary, our study provided evidence for the association between CSF‐cfDNA concentration and CNS‐IPI score, highlighting the importance of CSF‐cfDNA in the detection of CNS tumors in DLBCL. Five CSF‐CNS genes in CSF was found to be associated with CNS risk in DLBCL patients, which deserve further investigation to determine their relevance among the diagnosis, treatment, and outcome.

## FUNDING INFORMATION

The National Science Foundation of China; Grant Number: 81970176; Natural Science Foundation of Guangdong Province; Grant Number: 2019A1515011943.

## AUTHOR CONTRIBUTIONS

Study design: WXX, GY, and HHQ. Data acquisition: WXX, SCG, LMY, ARY, WXX, and CLB. Data analysis: WXX, HHX, GY, RY, and QO. Manuscript writing: WXX, HHQ, RY, XW, and YWS. Study supervision: HHQ.

## CONFLICT OF INTEREST

Ruoying Yu, Qiuxiang Ou, and Xue Wu are the shareholders or employees of Geneseeq Technology Inc. Canada. Xiaoxia Wang and Yang W. Shao are the shareholders or employees of Nanjing Geneseeq Technology Inc. The remaining authors have no conflict of interest to declare.

## Supporting information

SUPPORTING INFORMATION

SUPPORTING INFORMATION

## Data Availability

The datasets during and/or analyzed during the current study are available from the corresponding author on reasonable request.
