# Supplementary material for: Association of circulating tumor DNA from the cerebrospinal fluid with high‐risk CNS involvement in patients with diffuse large B‐cell lymphoma
Source: Clin Transl Med. 2021 Jan 15;11(1):e236. doi: 10.1002/ctm2.236 (PMC7809600; doi:10.1002/ctm2.236)
Supplement: Supplementary file 1 — SUPPORTING INFORMATION [file CTM2-11-e236-s001.docx]

**Methods**

**Patient**

In this retrospective study, a total of 67 patients with systemic DLBCL with high-risk for CNS involvement was recruited in the Sun Yat-sen University Cancer Center (Guangzhou, China) from November 2017 to September 2019. Patient consent form was obtained from each patient following the guideline of Institutional Review Board requirements and the Declaration of Helsinki. The study was approved by the Ethical Committee of the Sun Yat-sen University Cancer Center.

DLBCL subtype was determined using immunohistochemical staining for CD10, BCL-6, and MUM-1, following the Hans classification. The inclusion criteria for patients at high-risk for CNS involvement were as follows: CNS-IPI score 4-6, CNS-IPI score2-3, ABC type DEL+, testicular/kidney/adrenal involvement. Patient criteria was shown in Supplementary Table 1. Total 67 patients with DLBCL were assessed as high-risk for CNS. Tumor tissue, plasma and CSF were sampled from patients including before and after treatment time point. Among them, 53 patients had matched tissue, CSF and plasma samples. Of 53 patients with matched CSF samples, 20 patients with high-risk for CNS had cfDNA detected (CSF-cfDNA positive). CSF and plasma were collected at the same time from each patient.

Patients presented with CNS-IPI score 0-1, CNS-IPI score 2-3 without DEL+ABC- type, no testicular/Kidney/adrenal involvement were considered at low-risk for CNS relapse. Tumor tissue from low-risk group were sampled before treatment.

**DNA extraction, library preparation and target enrichment**

As previously described, a standardized approach was used to extract cfDNA from CSF and plasma as well as genomic DNA from FFPE^1,2^. CSF was obtained through lumbar puncture as a standard practice for DLBCL patients at high-risk for CNS involvement following National Comprehensive Cancer Network(NCCN) guidelines. Within four hours of collection, the cellular fraction was removed by two-step centrifugation at 4^o^C including 1,900g for 10mins then 16,000g for 10mins. Plasma samples were first separated with blood cell sediment after centrifuging whole blood samples at 4^o^C for 10min 1,600g, then recentrifuged at 4^o^C 16,000g for 10mins. Both cfDNA from CSF and plasma were extracted using Qiagen QIAmp Circulating Nucleic Acid Kit(Qiagen, Germany) following the manufacturer’s protocols. Genomic DNA from tumor tissue samples was extracted using QIAmp DNA FFPE Tissue Kit(Qiagen, Germany) following the manufacturer’s protocols. Normal tissue was obtained from each patient using oral swab(COPAN, USA) following the manufacturer’s protocols. Genomic DNA from normal tissue was extracted using DNeasy Blood&Tissue Kit (Qiagen, Germany) and used as a control to filter out germline mutations.

A targeted resequencing gene panel, including coding exons and splice sites of more than 400 genes that are recurrently mutated in B cell lymphoma, was specifically designed for this project. NGS tests were performed and underwent CLIA-certified and CAP-accredited assay validation at a centralized clinical testing center(Nanjing Geneseeq Technology, Inc, Nanjing, China). Libraries were sequenced on a HiSeq 4000 NGS platform (Illumina), and the sequencing data were analyzed to detect genomic alterations. The mean coverage depth was ~100X for controls, ~1,000X for tissues, and ~5,000X for cfDNA samples.

**Sequencing and data processing/Genomic analysis**

Sequencing data was processed as previously described^1^. In brief, the data was first demultiplexed and subjected to FASTQ file quality control to remove low quality data or N bases. Qualified reads were mapped to the reference human genome hg19 using Burrows-Wheller Aligner and Genome Analysis Toolkit (GATK 3.4.0) was employed to apply the local realignment around indels and base quality score recalibration. Picard was used to remove PCR duplicates. VarScan2 was employed for the detection of single-nucleotide variations (SNVs) and insertion/deletion mutations. The resulting mutation lists were further filtered through an internally collected list (1,000 normal samples) of recurrent artifacts on the same sequencing platform. Specifically, if a variant was detected (i.e. ≥3 mutant reads and ≥1% variant frequency) in >10% of the standard normal samples, it was considered a likely systematic artifact and was removed. Mutations were also removed if they were common variants present in >1% population frequency in the 1,000 Genomes Project (<https://www.internationalgenome.org>) or 65,000 exomes project (<http://exac.broadinstitute.org>).

ADTEx was used to identify copy number variations (CNVs) with a normal human DNA sample NA18535 with default parameters as previously described^1^. The cutoff of log2 ratio was set at ±0.6 for copy number changes (corresponding to 1.5-fold copy number gain and 0.65-fold copy number loss).

**Statistical analysis**

The sensitivity and specificity of CSF and plasma cfDNA genotyping were calculated in comparison with tumor gDNA genotyping as the gold standard. Significant factors in univariate analysis were further subjected to multivariate analysis using the Cox regression model to define the independent risk factors for survival rates. Two-sided P value < 0.05 was considered to represent a statistically significant difference. The analysis was performed with SPSS version 22.0. Pathway analysis was performed in R using KEGG database.

1. Yang, Z. *et al.* Investigating Novel Resistance Mechanisms to Third-Generation EGFR Tyrosine Kinase Inhibitor Osimertinib in Non-Small Cell Lung Cancer Patients. *Clin Cancer Res* **24**, 3097-3107 (2018).

2. Xing, L. *et al.* Biomarkers of osimertinib response in patients with refractory, EGFR-T790M-positive non-small cell lung cancer and central nervous system metastases:the APOLLO study. *Clin Cancer Res* (2020).
