# Supplementary material for: Association of circulating tumor DNA from the cerebrospinal fluid with high‐risk CNS involvement in patients with diffuse large B‐cell lymphoma
Source: Clin Transl Med. 2021 Jan 15;11(1):e236. doi: 10.1002/ctm2.236 (PMC7809600; doi:10.1002/ctm2.236)

**Table S1. Characteristics of 67 DLBCL patients with high risk for CNS.** LDH: lactate dehydrogenase. GCB: germinal center B-cell-like subtype.

| **DLBCL cohort** | **N=67** |
| --- | --- |
| **Age** | **N(%)** |
| Median, Range | 59,18~87 |
| ≥ 60 | 31(46.3%) |
| **Sex** | **N(%)** |
| Male | 46(68.7%) |
| Female | 21(31.3%) |
| **LDH** | **N(%)** |
| Elevated | 31(46.3%) |
| **IPI** | **N(%)** |
| 0–1 | 15(22.4%) |
| 2–3 | 39(58.2%) |
| 4–5 | 13(19.4%) |
| **CNS-IPI** | **N(%)** |
| 0–1 Low-risk | 14(20.9%) |
| 2–3 Intermediate-risk | 37(55.2%) |
| 4–6 High-risk | 16(23.9%) |
| **Extranodal sites** | **N(%)** |
| ≥2 | 48(71.6%) |
| **COO subtypes** | **N(%)** |
| GCB | 23(34.3%) |
| Non–GCB | 40(59.7%) |
| NA | 4(6.0%) |

Supplementary Figure 1. Patient criteria. CSF-cfDNA positive: samples with detectable extracted cfDNA. DLBCL: diffuse large B-cell lymphoma. CNS: central nervous system. CNS-IPI: central nervous system-International Prognostic Index. ABC:  activated B-cell like subtype. DEL+: have double-expressor lymphoma.


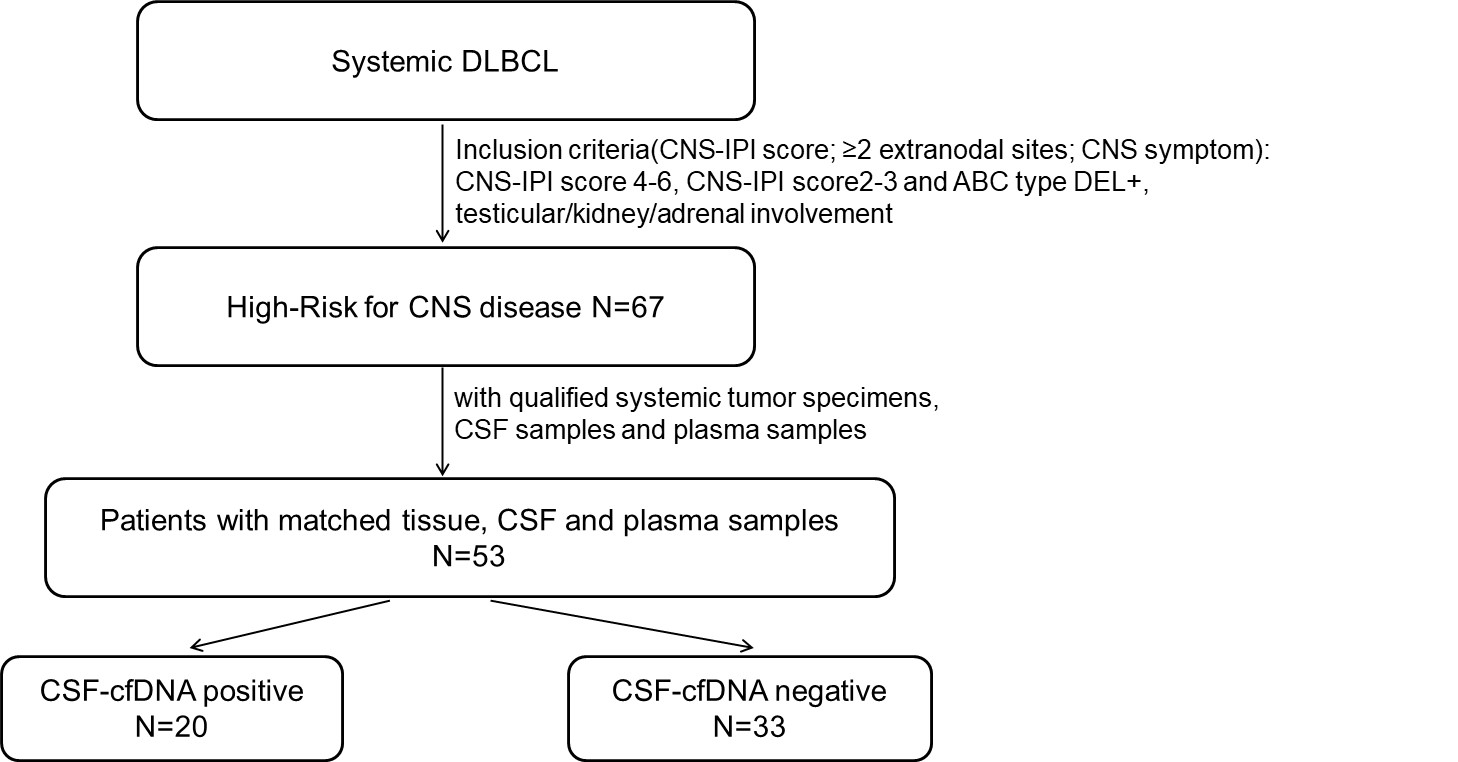


Supplementary Figure 2. Alterations in five CNS-related genes in DLBCL patients at low-risk and high-risk for CNS relapse. Patients presented with CNS-IPI score 0-1, CNS-IPI score 2-3 and not DEL+ABC- type, no testicular/Kidney/adrenal involvement were considered at low-risk for CNS relapse. Tumor tissue from low-risk group were sampled before treatment. (A) numbers of DLBCL patients with mutated five CNS-related genes in low-risk and high-risk group. (B) Numbers of mutated five CNS-related genes in low-risk and high-risk group. (C) Mutation frequency of altered genes in tumor tissue from DLBCL patients at low-risk and high-risk for CNS relapse. *: p＜0.05; **: p＜0.01; ***: p＜0.001.


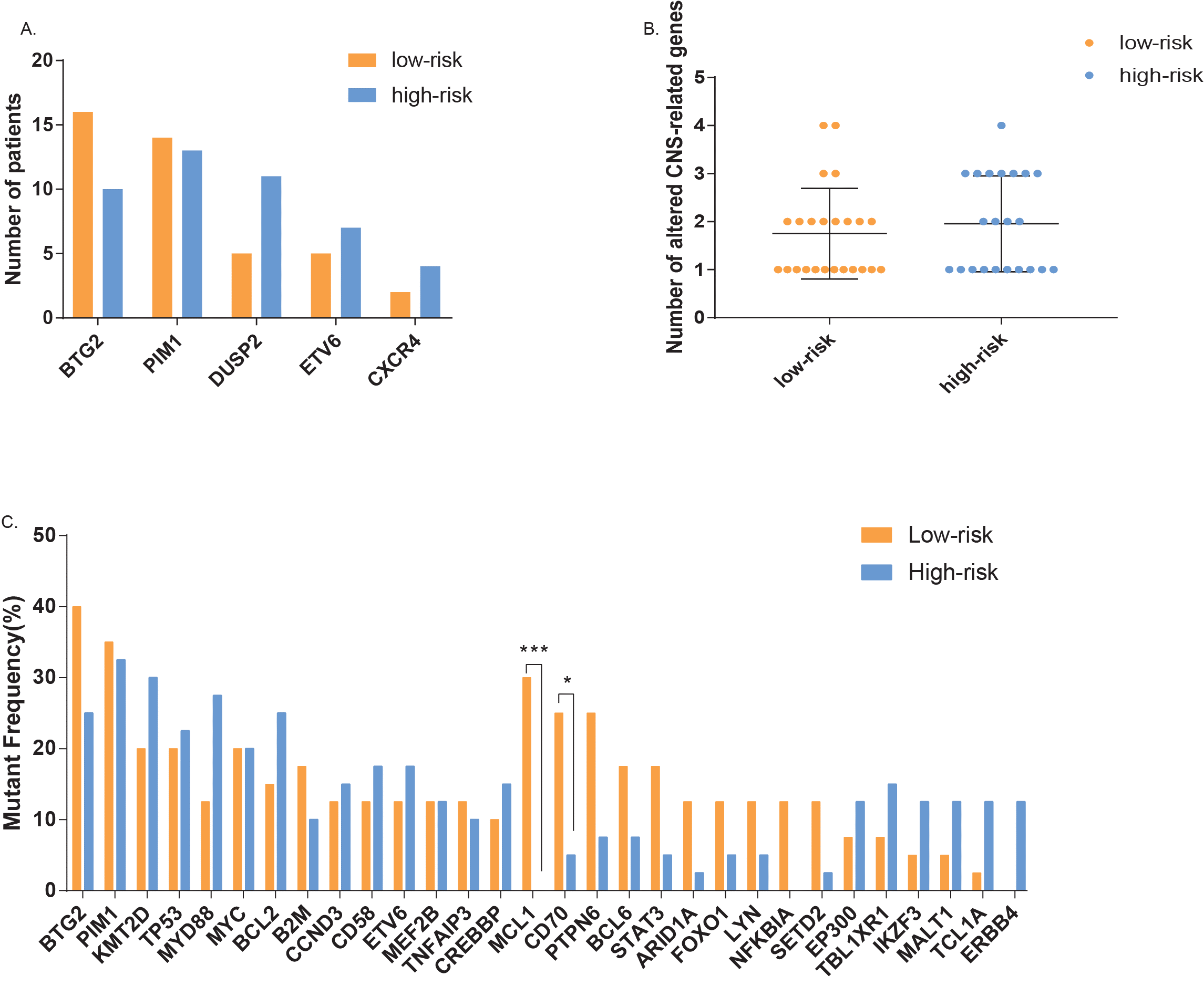

Supplement: Supplementary file 2 — SUPPORTING INFORMATION [file CTM2-11-e236-s002.docx]
